# Entrainment of the mouse circadian clock by sub-acute physical and psychological stress

**DOI:** 10.1038/srep11417

**Published:** 2015-06-15

**Authors:** Yu Tahara, Takuya Shiraishi, Yosuke Kikuchi, Atsushi Haraguchi, Daisuke Kuriki, Hiroyuki Sasaki, Hiroaki Motohashi, Tomoko Sakai, Shigenobu Shibata

**Affiliations:** 1 Laboratory of Physiology and Pharmacology, School of Advanced Science and Engineering, Waseda University, Tokyo, Japan

## Abstract

The effects of acute stress on the peripheral circadian system are not well understood *in vivo*. Here, we show that sub-acute stress caused by restraint or social defeat potently altered clock gene expression in the peripheral tissues of mice. In these peripheral tissues, as well as the hippocampus and cortex, stressful stimuli induced time-of-day-dependent phase-advances or -delays in rhythmic clock gene expression patterns; however, such changes were not observed in the suprachiasmatic nucleus, i.e. the central circadian clock. Moreover, several days of stress exposure at the beginning of the light period abolished circadian oscillations and caused internal desynchronisation of peripheral clocks. Stress-induced changes in circadian rhythmicity showed habituation and disappeared with long-term exposure to repeated stress. These findings suggest that sub-acute physical/psychological stress potently entrains peripheral clocks and causes transient dysregulation of circadian clocks *in vivo*.

The circadian clock system in mammals comprises endogenous pacemakers located in the brain and periphery that organise various biological activities, including behaviour, mood, memory, metabolism, body temperature, neural activity, and hormone release^1^,^2^. The suprachiasmatic nucleus (SCN) in the hypothalamus is the master pacemaker that regulates peripheral clocks in tissues through behavioural, endocrine, and neural pathways. Clocks are synchronised by environmental factors including light-dark cycles, food, exercise, and drugs^3^. At the molecular level, a feedback loop of transcriptional activation by CLOCK/BMAL1 and repression by PER/CRY complexes drives the rhythmic expression of these proteins in each cell over a ~24-h cycle. In addition, *Dbp* and *Rev-erbα* – encoding a transcriptional activator and repressor, respectively – also show oscillatory expression over periods of approximately 24 h and regulate clock-controlled output genes^4^.

External or internal stressors activate the physiological fight-or-flight system that maintains homeostasis in organisms^5^. In humans and rodents, repeated exposure to stress induces habituation, which enables the organism to cope with additional stressful stimuli^6^,^7^. However, excessive or repeated unpredictable stress disrupts homeostasis and can lead to mood or anxiety disorders, which are often associated with abnormalities in circadian rhythms in the sleep-wake cycle, body temperature, and blood melatonin levels^8^,^9^,^10^. The circadian clock regulates the stress response system: the hypothalamic-pituitary-adrenal (HPA) and sympathetic-adrenal-medullary (SAM) axes that are activated during acute stress^11^,^12^ show circadian rhythmicity^13^,^14^,^15^. Moreover, dexamethasone-induced activation of the HPA axis stimulates *Per1* and *Per2* expression *in vitro* and *in vivo*^16^,^17^; likewise, activation of the SAM axis by adrenaline or noradrenaline leads to up-regulation of *Per1* in the liver *via* cyclic adenosine monophosphate and mitogen-associated protein kinase signalling^18^,^19^. Several reports suggest that acute and chronic stress modulate the circadian clock. For example, behavioural rhythm in hamsters was entrained by restraint stress^20^,^21^, although the same was not observed in mice exposed to acute stress^22^. In a mouse model of depression, chronic mild stress caused abnormal rhythms of behaviour and body temperature, as well as abnormal clock gene expression in central and peripheral clocks^23^,^24^,^25^,^26^,^27^,^28^.

The findings of the aforementioned studies suggest that stress can act as an entrainment cue for the circadian system; however, how acute stress affects the rhythmic expression of clock genes in peripheral tissues remains unclear. In the present study, the effects of sub-acute stress on the phase and amplitude of clock gene expression were investigated by using *in vivo* bioluminescence monitoring^29^. We found that physical and psychological stressors such as restraint or social defeat are strong synchronisers of peripheral clocks with time-of-day dependency. This entrainment was abolished after adaptation to stressful stimuli by repeated exposure, suggesting that acute stressors transiently but potently entrain peripheral clocks.

## Results

### Daily restraint stress induces phase entrainment of circadian clocks in extra-SCN brain regions and in peripheral tissues

**Figure 1:**
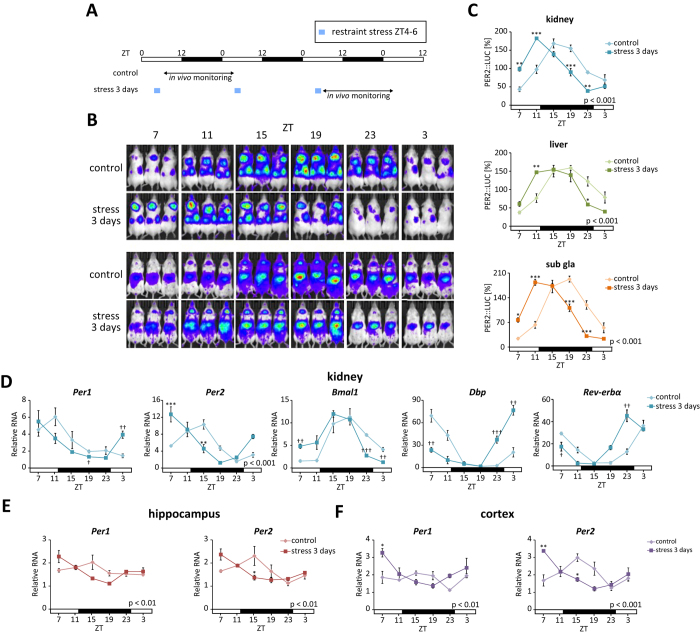
Restraint stress at ZT4–6 for 3 consecutive days causes phase-advance. (**A**) Experimental schedule. (**B**) Representative images of *in vivo* PER2::LUC bioluminescence in the kidney (upper panels) and in the liver and submandibular gland (sub gla) (lower panels). (**C**) Normalised PER2::LUC oscillations in control and stress groups. The number of tissues that met the criteria for rhythmicity is shown in Table S1. RNA expression of clock genes in the (**D**) kidney, (**E**) hippocampus, and (**F**) cortex (n = 3 at each time point; total n = 3 × 6 points for control and stress groups). Values are expressed as mean ± SEM. P value on the lower right side of each graph indicates results of a two-way ANOVA between the control and stress groups. *P < 0.05, ***P < 0.001 vs. control (two-way ANOVA with Tukey post-hoc test); ^†^P < 0.05, ^††^P < 0.01, ^†††^P < 0.001 vs. control (Student’s t test). Results of cosinor analysis of the RT-PCR data are described in Table S3.

**Figure 2:**
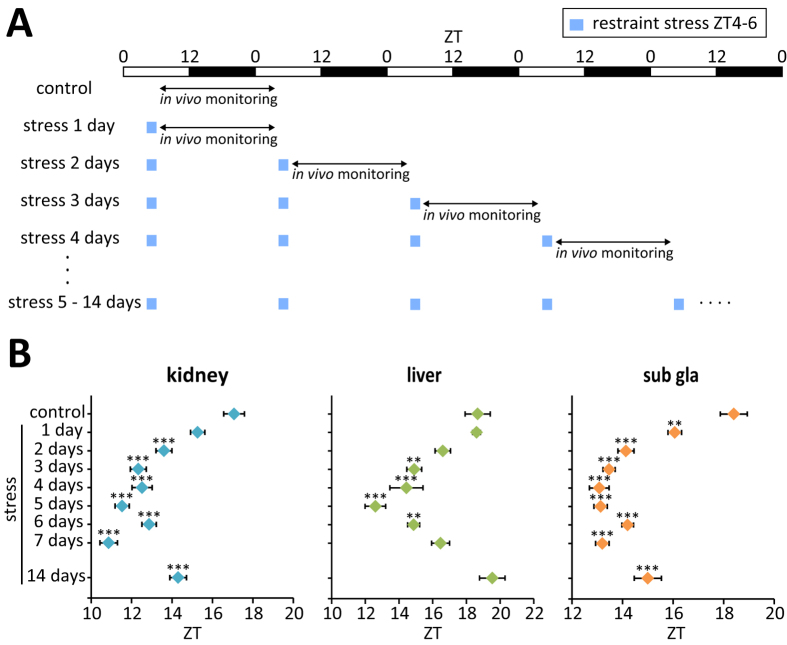
Long-term effects of restraint stress at ZT4–6 on peripheral PER2::LUC oscillations. (**A**) Experimental schedule. (**B**) Peak phases of peripheral PER2::LUC oscillations on each day. The number of tissues that met the criteria for rhythmicity is shown in Table S1. Values are expressed as mean ± SEM. **P < 0.01, ***P < 0.001 vs. control (one-way ANOVA with Dunnett post-hoc test).

A paradigm used for experiments on acute feeding entrainment^30^ was used to investigate the effects of stress on the entrainment of circadian clocks. Female PER2::LUC mice^31^ were restrained during the resting period – i.e. from Zeitgeber time (ZT)4–6 (lights were turned on at ZT0), for 3 consecutive days – and bioluminescence rhythms in their kidneys, livers, and submandibular glands were measured (Fig. 1A). The peak times of oscillatory PER2::LUC activity in each tissue were phase-advanced in stress-exposed mice compared with those in control mice (Fig. 1B,C). Rhythmic RNA expressions of the core clock genes *Per1*, *Per2*, *Bmal1*, *Dbp*, and *Rev-erbα* were also phase-advanced in these tissues as well as in the adrenal gland, indicating a systemic entrainment of circadian clocks to the stressful stimuli (Figs 1D, S1A, and Table S3). The phase shift values in PER2::LUC experiment were −4.7 h for the kidney, −3.7 h for the liver, and -5.0 h for the submandibular gland (Figs 1 and 2). The phase shift values in the *Per2* mRNA experiment were −5.2 h for the kidney, −4.0 h for the liver, and −3.2 h for the submandibular gland (Figs 1 and S1, Table S3).

Stress-induced phase changes in the kidney and submandibular gland were enhanced by additional application of daily stress over 4–5 days, and this effect persisted during 2 weeks of stress exposure (Fig. 2) suggesting that these clocks were synchronised to the external cue. However, in the liver, the phase gradually returned to normal over 6–7 days of stress exposure and returned to the starting value after 2 weeks (Fig. 2).

**Figure 3:**
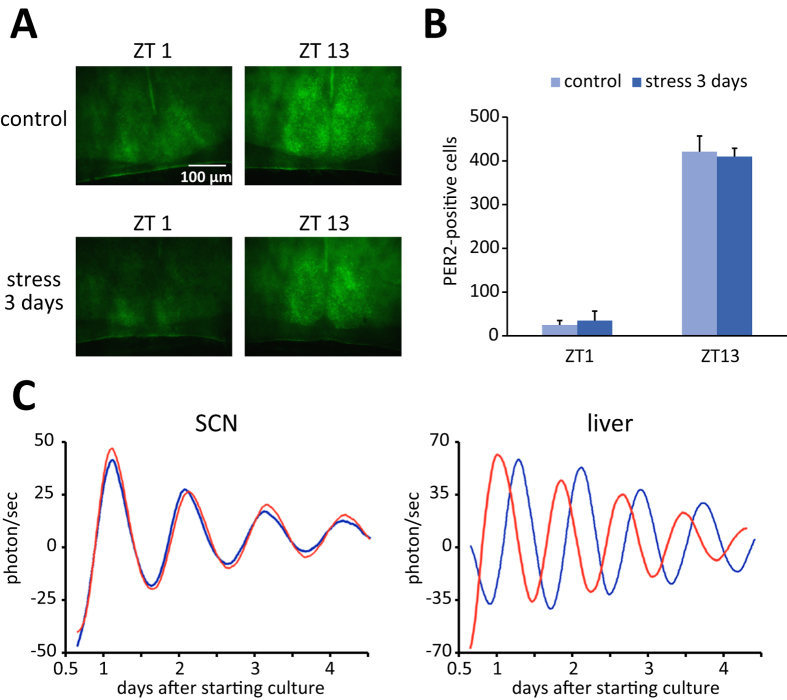
Effects of 3 days of restraint stress at ZT4–6 on the SCN. (**A**) Photomicrographs of PER2 immunofluorescence in the SCN of control mice or mice subjected to restraint stress (ZT4–6 for 3 days). (**B**) Quantitative analysis of PER2-positive cells. The number of mice was n = 4 both for the control and stress groups. Values are expressed as the mean + SEM. (**C**) Representative PER2::LUC bioluminescence rhythms from SCN and liver explants of control (blue line) or stressed (red line) mice. Waveforms were smoothened and detrended after measurement. The number of mice was n = 4 for the control group and n = 3 for the stress group.

As in the peripheral tissues, rhythmic *Per1* and *Per2* expression in the hippocampus and cortex was phase-advanced after 3 days of stress exposure (Fig. 1E,F, and Table S3). In contrast, PER2 levels in the SCN did not differ significantly between the control and stress treatment groups at ZT1 or ZT13 (Fig. 3A,B), as observed by immunohistochemistry. Additionally, bioluminescence oscillations in cultured SCN slices from PER2::LUC mice showed similar waveforms in the two groups (Fig. 3C; peak time of the first peak was 26.6 ± 0.5 h for the control and 26.1 ± 0.2 h for the stress treatment). Furthermore, similar results for bioluminescence from SCN slices were observed after the restraint stress at ZT0–2 or ZT12–14 (Figure S3). These results indicate that the central oscillator in the SCN was unaffected by stress and was not involved in the stress-induced phase entrainment of peripheral clocks. In contrast, liver explants from stress-treated mice showed striking phase alterations compared with those obtained from control mice, suggesting that the phase shift persisted *ex vivo* (Fig. 3C; peak time of the first peak was 30.4 ± 0.5 h for the control and 26.4 ± 1.0 h for the stress treatment; P < 0.05 by Student’s t test). However, the PER2::LUC oscillation in the liver returned to the normal phase 24 h after the 3-day stress exposure, whereas, in the kidney and submandibular gland, the phase was still advanced at this point in stressed mice compared with control mice (Figure S2). These experiments were conducted using female mice in order to eliminate the effects of stress arising from fighting among male mice; however, as in females, PER2::LUC oscillation in the peripheral tissues of male mice also showed significant phase-advance during 3 days of restraint stress (Figure S1B–S1D). Taken together, these data demonstrate that sub-acute restraint stress during daytime induces a phase-advance of circadian clocks in the peripheral tissues and the brain excluding the SCN.

### Stress-induced circadian changes show time-of-day dependence

**Figure 4:**
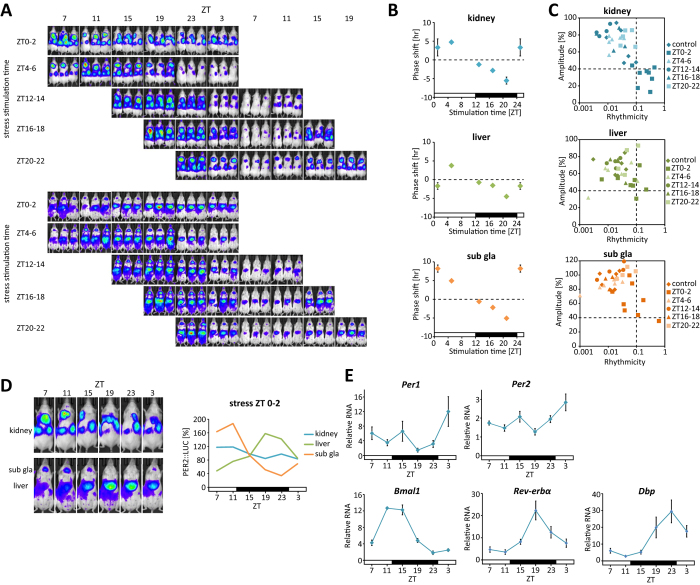
Time-of-day dependence of circadian changes in response to stress. (**A**) Representative images of *in vivo* PER2::LUC bioluminescence in the kidney (upper panels) and in the liver and submandibular gland (sub gla) (lower panels). The detailed experimental schedule is described in Supplementary Figure S4. (**B**) Phase-response curves of stress response in peripheral clocks. Increased and decreased phase shifts indicate phase-advance and -delay, respectively. Data for ZT25 were copied from ZT1. Graphs include all rhythmic and arrhythmic data because few tissues met the specified criteria at ZT0–2 in the stress group. (**C**) Correlation map of amplitude and rhythmicity evaluated by cosinor analysis. Data for individual mice are shown in the graphs. Broken lines indicate the cutoff value for rhythmicity. (**D**) Representative images of *in vivo* PER2::LUC bioluminescence (left) and normalised waveforms (right) after restraint stress at ZT0–2 for 3 days. (**E**) RNA expression profiles of clock genes in mice subjected to restraint stress at ZT0–2 for 3 days (n = 7 for each time point). Values are expressed as the mean ± SEM.

To examine the effects of restraint stress on the synchronisation of circadian clocks, phase response curves (PRC) were generated by analysing phase shift values of the stress response as a function of time of stimulation (detailed experimental schedules are shown in Figure S4). The intact data shown in Fig. 1 were used as the control because the timing of *in vivo* monitoring was not effective for the phase in bioluminescence rhythms^29^. The bioluminescence and phase shift values from the PRC suggested a time-of-day dependence in each tissue (Fig. 4A,B): stressful stimuli had no effect on the peripheral PER2::LUC oscillation when applied at ZT12–14 but induced a phase-delay at ZT20–22. Interestingly, stress at ZT0–2 produced internal desynchronisation of the PER2::LUC oscillation between tissues (Fig. 4A–D): the PER2::LUC phase was unaltered in the liver but became anti-phasic in the submandibular gland. Moreover, the PER2::LUC rhythm in the kidney was dampened or disappeared in individual mice, with the correlation map between amplitude and rhythmicity value revealing differences between control and stress-treated animals (Fig. 4C; P < 0.01, Fisher’s exact probability test). This was confirmed by measuring RNA expression patterns in the kidney after 3 days of restraint stress at ZT0–2. Rhythms of *Per1* and *Per2* expression were dampened, corresponding to the dampening of the PER2::LUC oscillation (Fig. 4E and Table S3). However, while *Bmal1*, *Rev-erbα*, and *Dbp* expression had day-night rhythms, the phases were advanced relative to that of control mice (Figs 1D, 4E, and Table S3), which suggests that stress-induced arrhythmicity was sensitive to *Per1/2*. Thus, restraint stress-induced changes in PER2::LUC oscillation show time-of-day dependence, with stress applied at the onset of the inactive period leading to internal desynchronisation of circadian rhythmicity.

### Stress-induced entrainment shows habituation after repeated, intermittent application of the stressor

**Figure 5:**
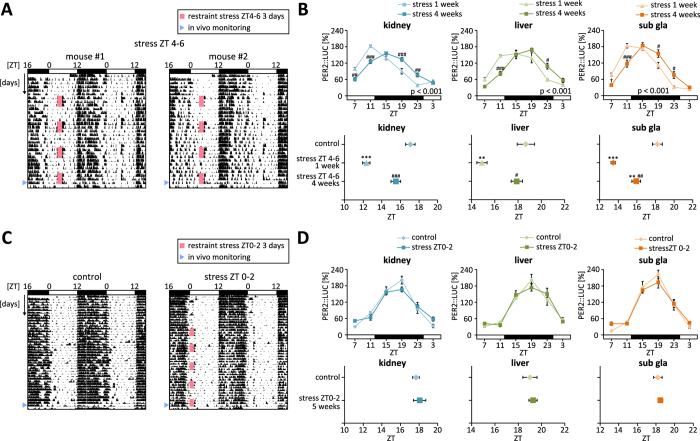
Effects of chronic, intermittent restraint stress on the peripheral clock. Mice were subjected to 3 days of restraint stress at ZT4–6 (A, B) or ZT0–2 (**C**,**D**) on Monday, Tuesday, and Wednesday for 4 weeks (**A**,**B**) or 5 weeks (C, D). On the final day of stress exposure, PER2::LUC rhythm was measured starting at ZT7. (**A**) Representative locomotor activity profiles of two stressed mice. (**B**) Waveforms (upper) and peak phases (lower) of PER2::LUC rhythm in each tissue. Data for control and 1 week of stress are the same as shown in Fig. 1. (**C**) Representative locomotor activity profiles for control and stressed mice. (**D**) Waveforms (upper) and peak phases (lower) of PER2::LUC oscillations in each tissue. Values are expressed as the mean ± SEM, and the P value on the lower right side of each graph indicates the results of a two-way ANOVA between the 1- and 4-week stress groups. *P < 0.05, **P < 0.01, ***P < 0.001 vs. control; ^#^P < 0.05, ^##^P < 0.01, ^###^P < 0.001 vs. stress for 1 week (two-way ANOVA with Tukey post-hoc test).

Physiological responses decline with repeated exposure to the same stressor^6^. To determine whether habituation occurred for stress-induced changes in the phase of PER2::LUC oscillation, mice were subjected to restraint stress from ZT4–6 on 3 consecutive days per week for 4 weeks (Fig. 5A,B). After the final round of intermittent stress exposure, PER2::LUC bioluminescence in peripheral tissues was measured. In the kidney and liver, the phase-advance observed during the 3-day period of restraint diminished after 4 weeks of exposure to the stimulus. In the submandibular gland, the phase-advance was less pronounced after 4 weeks than after the first 3 days of exposure to the stressful stimulus. After 2 h of restraint stress, corticosterone release in serum was also reduced in mice that were repeatedly stressed compared with mice subjected to a single 3 days period of stress (Figure S6A). Similarly, the desynchronisation observed following 3 days of stress was not detected after stress was applied at ZT0–2 for 5 weeks in the intermittent stress paradigm (Figs 4, 5C,D). These results indicate that habituation inhibits the stress-induced phase shift in peripheral clocks.

### The accuracy and mechanism of stress-induced entrainment

Long-term sleep deprivation can alter the circadian clock^32^,^33^; therefore, we investigated whether sleep interruption contributes to the restraint stress-induced changes in the circadian phase of peripheral clocks. Mild sleep deprivation caused by a cage change^34^ during ZT4–6 for 3 consecutive days did not shift the phase of peripheral clocks (Figure S7A), indicating that sleep deprivation was not involved in stress-induced circadian entrainment. Stress-induced changes in feeding behaviour and body temperature can also lead to alterations in circadian rhythm^35^,^36^,^37^,^38^,^39^,^40^. Mice subjected to scheduled feeding during the dark period (ZT13–17), which inhibited feeding behaviour after restraint stress, showed a clear phase-advance in peripheral clocks in conjunction with 3 days of restraint stress (Figure S7B and S7C). Body temperature increased (+1.5 °C) in accordance with heightened activity following restraint stress (Figs 5A and S7D); however, the increase was not sufficient to entrain peripheral clocks because cage change stimulation also caused a similar increase in body temperature^40^. These results indicate that feeding behaviour and body temperature were not involved in stress-induced phase changes in peripheral clocks.

**Figure 6:**
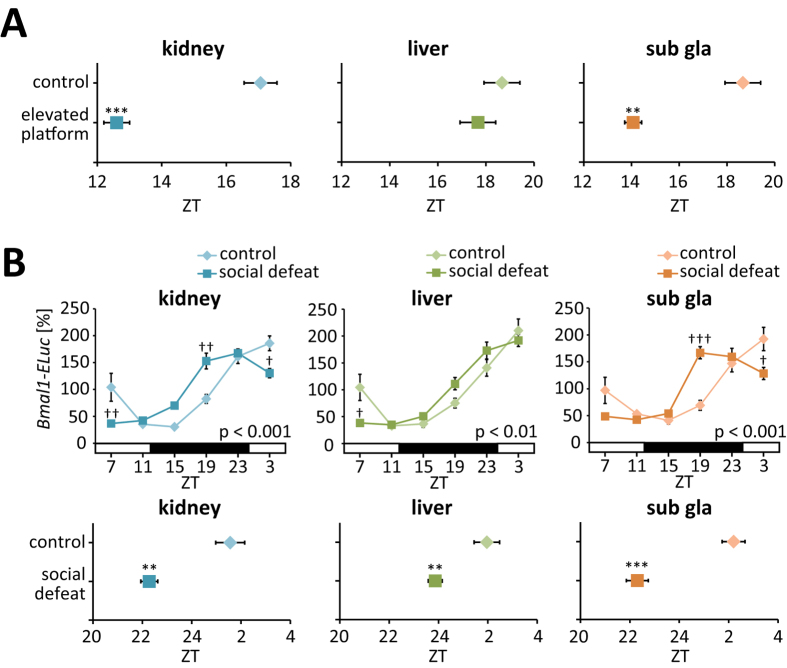
Effects of other stressors on peripheral clock entrainment. (**A**) Peak phases of PER2::LUC oscillation in each tissue after elevated platform stress at ZT4–6 for 3 days. (**B**) Normalised waveforms (upper) and peak phases (lower) of *Bmal1-ELuc* oscillations in each tissue after social defeat stress at ZT4–6 for 3 days. Values are expressed as the mean ± SEM. The P value on the lower right side of each graph indicates the results of the two-way ANOVA between control and stress groups. ^†^P < 0.05, ^††^P < 0.01, ^†††^P < 0.001 vs. control (two-way ANOVA with Tukey post-hoc test); **P < 0.01, ***P < 0.001 vs. control (Student’s t test). The number of tissues that met the criteria for rhythmicity is shown in Table S1.

To clarify the mechanisms underlying the stress-induced alterations, other types of stressors were administered to PER2::LUC or *Bmal1-ELuc* mice^29^,^41^. Mice placed on an elevated stage during ZT4–6 for 3 days showed notable entrainment of kidney and submandibular gland circadian clocks (Fig. 6A). Social defeat stress^42^, which was produced by 10 min of direct interaction followed by 110 min of indirect interaction between a male intruder *Bmal1-Eluc* mouse and a resident ICR mouse at ZT4–6 over 3 consecutive days, caused clear phase-advance of bioluminescence rhythms in the kidneys, livers, and submandibular glands of the intruder mice (Fig. 6B). Phase shifting of peripheral clocks was also induced by restraint stress in male *Bmal1-ELuc* mice (Figure S5). These other stressors also up-regulated the serum corticosterone levels in mice (Figure S6). In contrast, increased activity of the SAM axis by restraint stress was also confirmed in this study by measurement of catecholamine contents in peripheral tissues (Figure S6). In addition, injection of dexamethasone, norepinephrine, or adrenaline at ZT4 for 3 consecutive days also caused phase-advance of bioluminescence rhythms in each peripheral tissue (Figure S6). These results suggest that not only physical but also psychological stress has the capacity to entrain circadian clocks, and that the HPA and/or SAM axes may be involved in the mechanism of stress-induced entrainment.

## Discussion

This study provides the first *in vivo* demonstration of sub-acute external stress acting as a synchroniser of peripheral circadian clocks in mice. This entrainment was observed even during normal light-dark cycles, suggesting that it is more potent than light-induced entrainment. The PRC of this entrainment indicates that the advance, delay, or desynchronisation of circadian oscillation is dependent on the time of day. The phase-advance in PER2::LUC oscillations in the kidney and submandibular gland persisted after 2 weeks of restraint stress (Fig. 2), suggesting that peripheral clocks were completely entrained by chronic stress. Furthermore, clock gene expression in the kidneys, liver, and submandibular glands, as well as in the hippocampus and cortex, was entrained by the stressful stimuli, indicating that the effect was systemic. However, the central clock in the SCN was not entrained by the stress stimuli. This result is consistent with a previous report that showed the SCN does not respond to dexamethasone stimulation because of its lack of glucocorticoid receptors^16^. Stress induced entrainment might occur through the HPA and/or SAM axes because stress-induced corticosterone and adrenaline/noradrenaline reportedly act as synchronisers of circadian clocks^16^,^17^,^18^,^19^, and we confirmed that injection of these hormones or catecholamines caused phase entrainment in mice (Figure S6). However, we found that increased activity, sleep loss, and increased body temperature were not involved in this entrainment (Figure S7). Similar to restraint, stress from social defeat and placement on an elevated platform produced effects on the peripheral clocks, indicating that physical and psychological stressors are equally potent synchronisers.

Tissue specific responses to stress were observed in this study. In the liver, the phase-advance was lost after 2 weeks of restraint stress at ZT4–6 (Fig. 2), and no phase shift occurred when restraint stress was administered at ZT0–2 (Fig. 4). A possible reason for this finding is that the circadian clock in the liver is more strongly entrained by the feeding cycle than by stress. One study reported that the kidney was entrained by an anti-phasic corticosterone injection, whereas the liver clock followed a scheduled feeding cycle^43^. In the present study, 3 days of restraint stress phase-shifted PER2::LUC oscillation in the liver under normal and scheduled feeding conditions. Thus, although stress can modulate circadian rhythm in the liver, the effect may be abrogated by the more potent signal induced by feeding. In addition, the level of serum corticosterone was correlated with the entrainment of the liver clock. Compared with corticosterone released after restraint stress, the lower release of corticosterone following elevated platform stress or 4-week intermittent stress did not affect the phase of the liver clock (Figure S6). Taken together, our results suggest that the circadian clocks in the kidneys and submandibular glands are more responsive to stress than the clock in the liver.

An interesting finding of the present study was that restraint stress at ZT0–2 abolished PER2::LUC oscillations in the kidney. The stress-induced loss of circadian rhythm is referred to as singularity behaviour in chronobiology^44^,^45^,^46^. In mammals, bright light stimulation at a specific time in the circadian cycle causes arrhythmicity and loss of clock gene oscillation in the SCN^45^. The proposed explanation for singularity is that a potent entraining stimulus delivered at the critical transition from phase-delay to -advance causes desynchronisation of individual cellular clocks^45^,^47^. Here, we report for the first time singularity behaviour in mammalian peripheral *Per1* and *Per2* genes in response to restraint stress, which occurred in the kidneys of mice at the ZT0–2 time point in the PRC (Fig. 4B). In addition, internal desynchronisation was observed in circadian clocks of peripheral tissues in mice subjected to stress at ZT0–2 (Fig. 4D), as well as between the SCN and peripheral tissues following stress at ZT4–6 (Figs 1 and 3). This finding has medical implications: internal desynchronisation is considered jet lag, which is associated with a misalignment of the SCN and peripheral tissue clock phases^48^ and can lead to health problems^1^,^2^.

In summary, stressful stimuli had no effect on peripheral clocks when it was administered at the beginning of the active period but caused abnormal phase shifts or the loss of peripheral clock oscillations at other time points. Thus, environmental stressors in the evening or during the night can affect the circadian clock system. As for bright light or chrono-nutritional therapy^3^,^49^, controlling the HPA and SAM axes with mild stressors such as exercise^12^ could result in a mild entraining signal that acts on the circadian clock.

## Methods

### Animals

Heterozygous PER2::LUC knock-in mice^31^ on the ICR background, *Bmal1-ELuc* mice^41^ on the BALB/c background, and ICR (CD-1) mice (Tokyo Laboratory Animals Science Co., Ltd., Tokyo, Japan) were used in this study. The procedures conformed to the “Fundamental Guidelines for Proper Conduct of Animal Experiment and Related Activities in Academic Research Institutions” (published by the Ministry of Education, Culture, Sports, Science and Technology, Japan) and were approved by the Committee for Animal Experimentation of the School of Science and Engineering at Waseda University (permission #2013-A061). Mice (2–6 months old) were maintained on a 12:12 light/dark cycle (with lights on at 08:00 h) at room temperature (23 °C ± 0.5 °C) and were provided with a standard MF diet (Oriental Yeast Co., Ltd., Tokyo, Japan) and water *ad libitum*. The number of mice used in each experiment is shown in Table S1.

### Application of stressful stimuli

Mice were subjected to restraint stress using a wire-mesh bag (3 × 6 × 12 cm) clipped to their home cage. Individually housed mice were sleep-deprived by exchanging the bedding in their cages with new fresh wood shavings every 30 min for 2 h, after which the mice were returned to their original cages^34^. Elevated platform stress was administered by placing individual mice on a small stage (10 × 10 cm) at a height of 50 cm from the floor. *Bmal1-ELuc* intruder mice were subjected to social defeat stress by transferring them to cages of ICR mice (one intruder per resident mouse)^42^. Mice were allowed free contact with each other for the first 10 min; a wire mesh was then placed between them for 110 min to prevent fighting while still permitting interaction, after which intruders were returned to their home cages.

### *In vivo* recording of bioluminescence rhythm in peripheral tissues

Bioluminescence oscillations in peripheral tissues were monitored as previously described^29^. Briefly, mice were anaesthetised with a mixture of isoflurane (Mylan Inc., Tokyo, Japan) and concentrated oxygen. d-luciferin potassium salt (Promega, Madison, WI, USA) was injected subcutaneously (15 mg/kg) into the back near the neck. Images were acquired using an *in vivo* imaging system (Perkin Elmer, Waltham, MA, USA) with a 1-min exposure time from the dorsal- and ventral-up positions at 8 min and 10 min after luciferin injection, respectively. Images were obtained six times a day at 4-h intervals. Mice were returned to their home cages between imaging sessions. Photon counts for each tissue were analysed using Living Image 3.2 software (Perkin Elmer). The average photon/s value for the six time points on each day was designated as 100%, and bioluminescence rhythm for the entire day was expressed as a percentage of each set of six time points for individual organs. The peak phase, amplitude, and rhythmicity of normalised data were determined using the single cosinor procedure program (Acro.exe version 3.5)^50^. Cutoff values for amplitude (>40%) and rhythmicity (<0.1) were established to determine whether data were rhythmic or arrhythmic, and only rhythmic data were used for analyses of peak phase and average waveforms of normalised PER2::LUC rhythms. Tissue samples that met these criteria are shown in Table S1.

### *Ex vivo* recording of bioluminescence rhythm from tissue slices

An *ex vivo* luciferase assay was used to record bioluminescence rhythm in tissue slices^29^. SCN samples from PER2::LUC mice were sectioned at a thickness of 300 μm on a DTK-1500 vibratome (D.S.K., Kyoto, Japan). SCN sections were placed on a Millicell-CM culture insert (Merck Millipore, Billerica, MA, USA) and, in 35-mm Petri dishes (AGC Techno Glass Co. Ltd., Tokyo, Japan), liver explants were cultured in 1.3 ml Dulbecco’s modified Eagle medium (Life Technologies, CA, USA) with d-luciferin potassium (Promega). Cultures were incubated at 37 °C, and bioluminescence was monitored for 1 min at 10-min intervals using a LumiCycle luminometer (Actimetrics, Wilmette, IL, USA).

### Real-time RT-PCR

RNA was extracted from tissues using phenol (Omega Bio-Tek Inc., Norcross, GA, USA) for peripheral tissues or Trizol (Life Technologies) for brain tissue. Real-time reverse transcription PCR was performed using the One-Step SYBR RT-PCR Kit (Takara Bio Inc., Shiga, Japan) with specific primer pairs (listed in Table S4) on a Piko Real PCR system (Thermo Fisher Scientific, Waltham, MA, USA). Primers were designed using Primer 3 software^51^,^52^. The relative expression levels of target genes were normalised to that of *Gapdh*. Data were analysed using the ΔΔCt method. A melt curve analysis in each primer was performed to identify non-specific products.

### Immunohistochemistry

Brains were dissected and fixed with 4% paraformaldehyde at ZT13 and ZT1 after 3 days of restraint stress (ZT4–6), and then transferred into 20% sucrose solution and stored overnight at 4 °C. Sections were cut at a thickness of 20 μm on a cryostat (Leica Microsystems, Wetzlar, Germany), stained with anti-PER2 antibody (1:1000; Alpha Diagnostic International, San Antonio, TX, USA) overnight at 4 °C, and prepared for visualisation by incubation with an anti-rabbit IgG Alexa Flour488 secondary antibody (1:1000; Cell Signaling Technology, Danvers, MA, USA) and DAPI (Vector Laboratories, Burlingame, CA, USA) for 1 h at room temperature. Sections were imaged using a BZ-8100 fluorescence microscope (Keyence, Osaka, Japan) with 20× objective and 10× ocular lenses. Positive cells were counted using Image J software (National Institutes of Health, Bethesda, MD, USA), and the sum of all positive cells from the bilateral SCN in one slice per mouse was recorded.

### Statistical analysis

Data were analysed using GraphPad Prism (version 6.03, GraphPad software, San Diego, CA, USA). Equal variance and normal distribution tests were performed to select the appropriate statistical approach. Parametric analyses were conducted using a one-way, two-way, or two-way repeated measures ANOVA with Tukey, Dunnett, or Student’s *t*-tests for post-hoc analysis. Non-parametric analysis was carried out using a Kruskal-Wallis/Friedman test with a Dunn or Mann-Whitney test for post-hoc analysis. For the statistical analysis of RT-PCR time series data, once the data had passed equal variance and normal distribution tests, and if it could not be analysed by two-way ANOVA, Student’s *t*-tests were conducted to test for differences between two groups at specific time points. F and P values from one- or two-way ANOVA analyses are shown in Table S2. Data are expressed as the mean ± SEM. P < 0.05 was considered statistically significant.

## Additional Information

**How to cite this article**: Tahara, Y. *et al.* Entrainment of the mouse circadian clock by sub-acute physical and psychological stress. *Sci. Rep.* **5**, 11417; doi: 10.1038/srep11417 (2015).

## Supplementary Material

Supplementary Information
