# Supplementary material for: Entrainment of the mouse circadian clock by sub-acute physical and psychological stress
Source: Sci Rep. 2015 Jun 15;5:11417. doi: 10.1038/srep11417 (PMC4466793; doi:10.1038/srep11417)
Supplement: Supplementary Information [file srep11417-s1.pdf]

Supplementary Information

Entrainment of the mouse circadian clock by sub-acute physical and psychological stress

Yu Tahara[1], Takuya Shiraishi[1], Yosuke Kikuchi[1], Atsushi Haraguchi[1], Daisuke Kuriki[1], Hiroyuki Sasaki[1], Hiroyuki Motohashi[1], Tomoko Sakai[1], and Shigenobu Shibata[1,*]

[1]Laboratory of Physiology and Pharmacology, School of Advanced Science and Engineering, Waseda University, Tokyo, Japan

*Correspondence and proofs should be sent to:
Shigenobu Shibata
Laboratory of Physiology and Pharmacology, School of Advanced Science and Engineering
Waseda University
Wakamatsu-cho 2-2, Shinjuku-ku
Tokyo 162-8480, Japan
Telephone: +81-3-5369-7318; Fax: +81-3-3341-9815; E-mail: shibatas@waseda.jp

Supplemental methods

ELISA

Whole blood was allowed to clot by leaving it at room temperature for over 30 min, and then clots were removed by centrifuging at 3000 rpm and at room temperature for 20 min. Sampling time is described in the Figure S6 legend. An ELISA (Assay Pro, MO, USA) was used to measure serum corticosterone levels according to the manufacturer's instructions.

HPLC

Tissue monoamine content was measured by HPLC-ECD (HTEC 500) (Eicom, Kyoto, Japan). At ZT4.5, untreated mice (intact) and mice treated with restraint stress (ZT4–4.5) were deeply anaesthetized with isoflurane and their livers, kidneys, and submandibular glands were removed. To each tissue sample, we added 0.2 mol/l perchloric acid (including 100 μM EDTA・2Na) and 20 ng of isoproterenol. Samples were homogenized using a micro homogenizer, and then centrifuged at 15,000 *g* and 4°C for 15 min. The supernatants were collected for each sample, and these were filtered using a 0.45-μm filter. The quantity of monoamine in each 20-μl sample was measured by using HPLC-ECD and the following conditions: the transfer phase consisted of a) 85 % of 0.1 mol/l acetate citric acid buffer (pH 3.5) including 5 mg/l EDTA・2Na and 190 mg/l 1-octanesulfonic acid sodium salt and b) 15 % methanol (99 % purity); the velocity of the flow was 500 μL/min; the column temperature was set to 25°C; the applied voltage was set to +750 mV vs. Ag/AgCl. The data were analyzed with EPC-300 software (Eicom). Epinephrine could not be detected in the submandibular gland (sub gla).

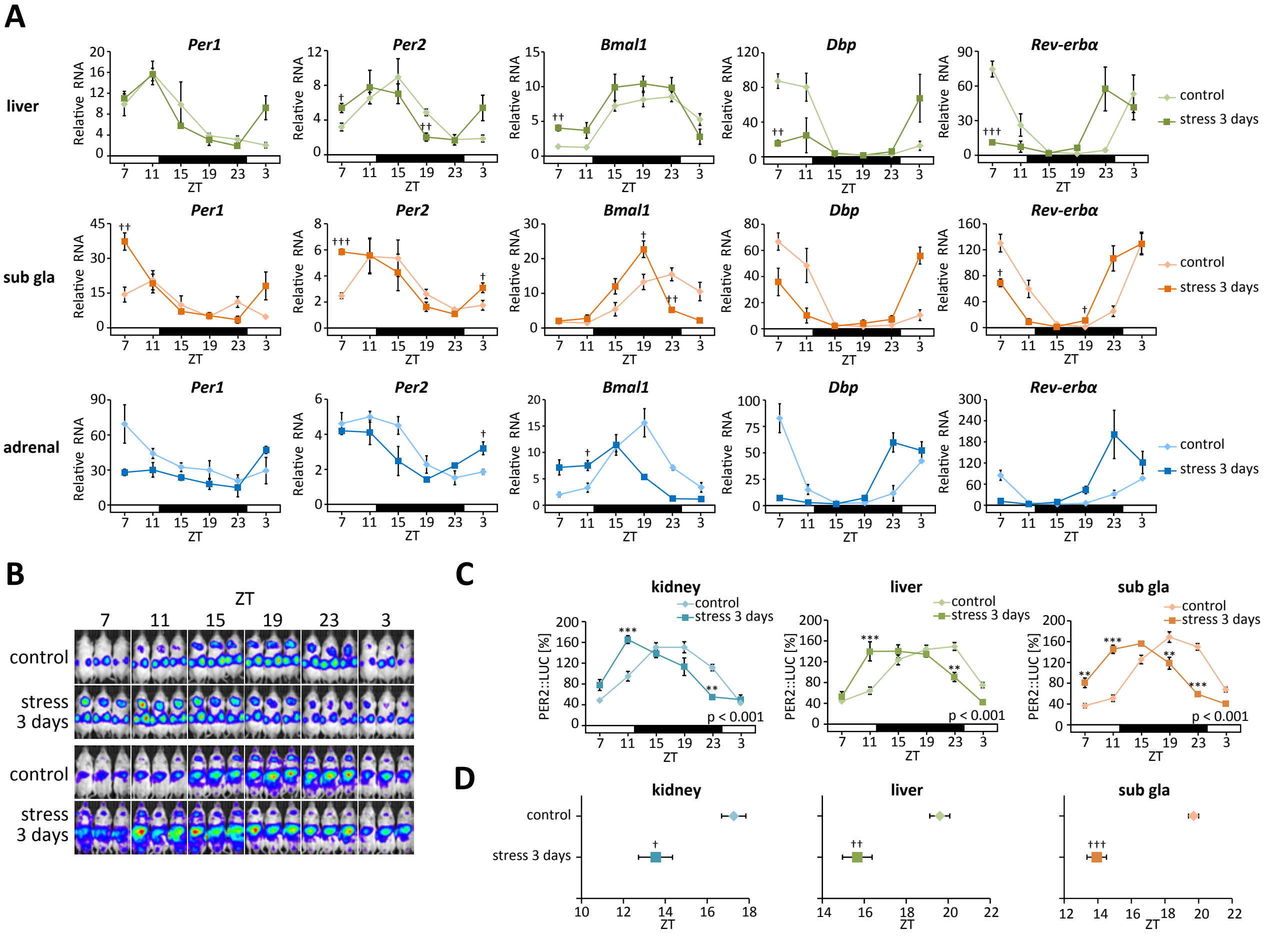

Supplementary Figure S1. Restraint stress at ZT4–6 for 3 consecutive days causes phase-advance of clock gene expression rhythms in the brain and peripheral tissues. (A) mRNA expression profiles of clock genes in the liver, submandibular gland (sub gla), and adrenal gland in control mice and mice subjected to restraint stress for 3 days at ZT4–6 (n = 3 for each time point). (B–D) Effects of 3 days of restraint stress at ZT4–6 on peripheral clocks in male PER2::LUC mice. Representative images of *in vivo* PER2::LUC bioluminescence in the kidney (B, upper panels) and in the liver and submandibular gland (D, lower panels) are shown. Waveforms (C) and peak phases (D) of PER2::LUC oscillations in each tissue are also shown. Values are expressed as mean ± SEM. P values shown on the lower right side of each graph indicate results from a two-way ANOVA between the control and stress groups. $^{**}P < 0.01$, $^{***}P < 0.001$ vs. control (two-way ANOVA with Tukey post-hoc test); $^{\dagger}P < 0.05$, $^{\dagger\dagger}P < 0.01$, $^{\dagger\dagger\dagger}P < 0.001$ vs. control (Student's

t-test). The number of tissues that met the criteria for rhythmicity is shown in Table S1.

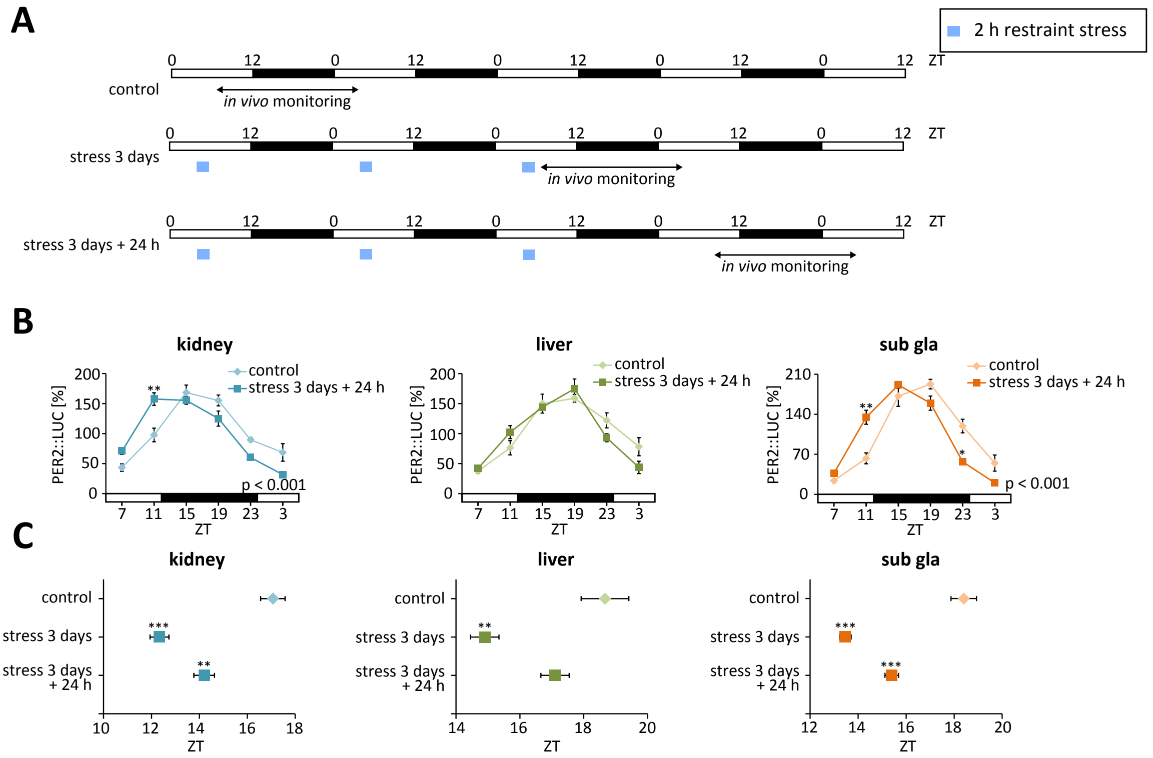

Supplementary Figure S2. Phase-advance is maintained in peripheral clocks 24 h after restraint stress at ZT4–6 for 3 days.

Mice were subjected to 3 days of restraint stress at ZT4–6, and PER2::LUC oscillations were measured starting from ZT7 on the day after the third session of stress exposure. Experimental schedule (A), waveforms (B), and peak phases (C) of PER2::LUC oscillations in each tissue are shown. Values are expressed as mean ± SEM. P values shown on the lower right side of each graph indicate results from a two-way ANOVA between the control and stress groups. $^{**}P < 0.01$, $^{***}P < 0.001$ vs. control (two-way ANOVA with Tukey post-hoc test). The number of tissues that met the criteria for rhythmicity is shown in Table S1.

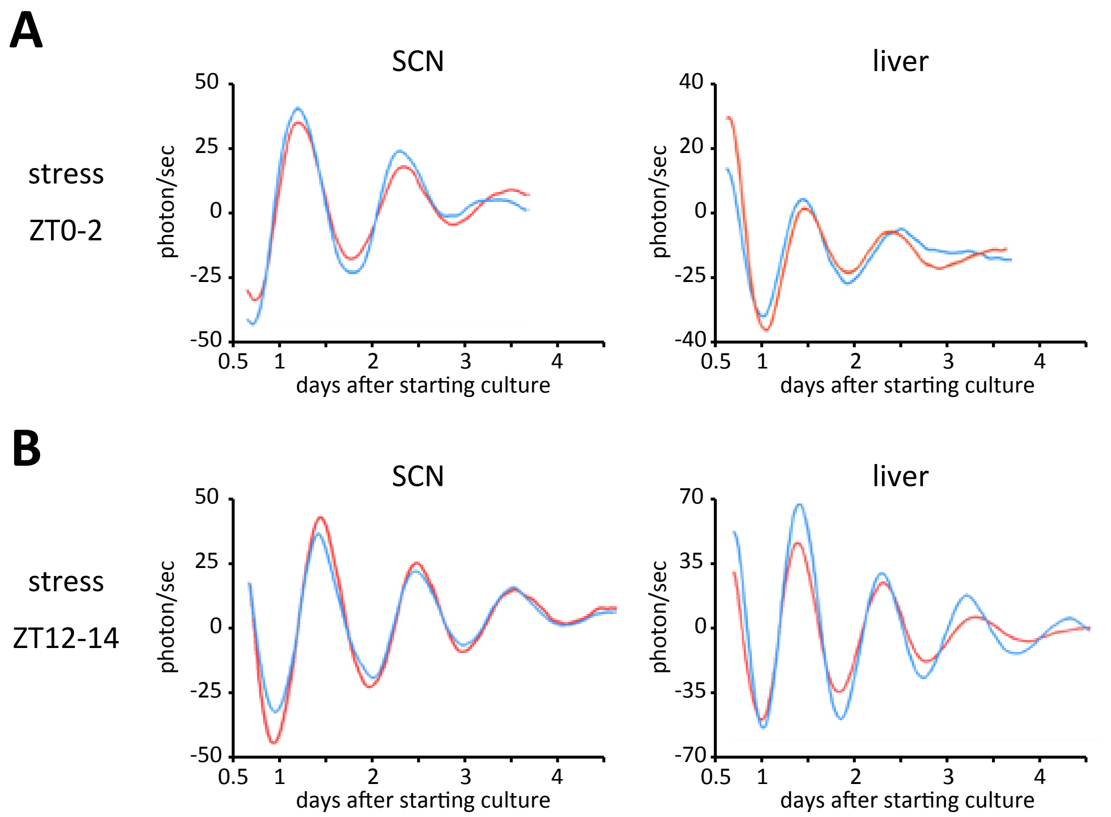

Supplementary Figure S3. Effects of 3 days of restraint stress at ZT0–2 or ZT12–14 on the SCN and liver.

PER2::LUC bioluminescence rhythms from SCN and liver explants of control (blue line) or stressed (red line) mice. Tissues were extracted at ZT5–6 and ZT1–2 after the final stress stimuli in each group, i.e. (A) stress ZT0–2 and (B) stress ZT12–14, respectively. Waveforms were smoothened and detrended after measurement. The number of mice was n = 4 for both the control group and the stress group in each experiment. The peak time of the first peak in (A) was 25.9 ± 0.5 h for the control group and 25.7 ± 0.8 h for the stress group in the SCN, and 30.5 ± 0.5 h for the control group and 31.2 ± 0.4 h for the stress group in the liver. The peak time of the first peak in (B) was 32.9 ± 0.4 h for the control group and 32.9 ± 0.7 h for the stress group in the SCN, and 33.9 ± 0.6 h for the control group and 34.2 ± 0.3 h for the stress group in the liver.

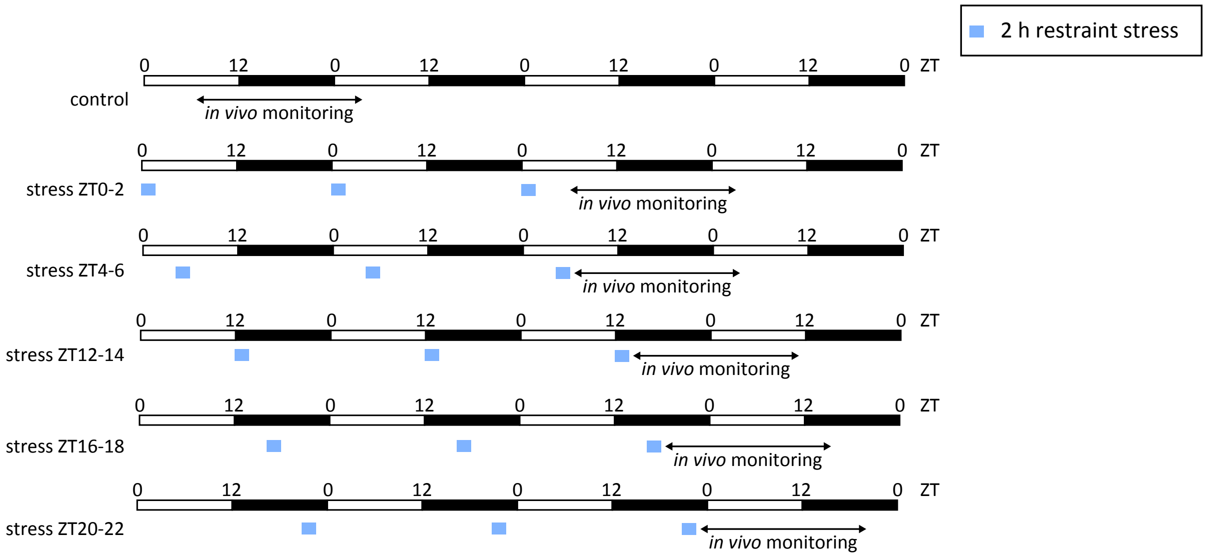

Supplementary Figure S4. Experimental schedule of restraint stress at different time points.

White and black bars indicate light and dark periods, respectively. Numbers on the white and black bars indicate ZT.

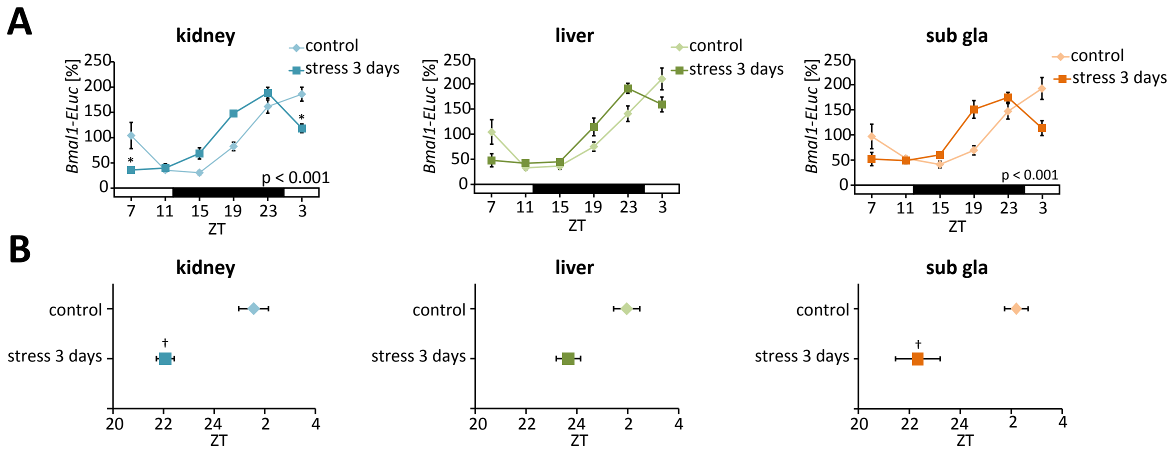

Supplementary Figure S5. Effects of restraint stress at ZT4–6 on peripheral *Bmal1-ELuc* rhythms.

Male *Bmal1-ELuc* mice were subjected to restraint stress at ZT4–6 for 3 consecutive days, after which *Bmal1-ELuc* rhythm was measured starting from ZT7 on the final day of stress exposure. Waveforms (A) and peak phases (B) of peripheral clocks are shown. Values are expressed as mean ± SEM. P values shown on the lower right side of each graph indicate results from a two-way ANOVA between the control and stress groups. *P < 0.05 vs. control (two-way ANOVA with Tukey post-hoc test); †P < 0.05 vs. control (Mann-Whitney test). The number of tissues that met the criteria for rhythmicity is shown in Table S1.

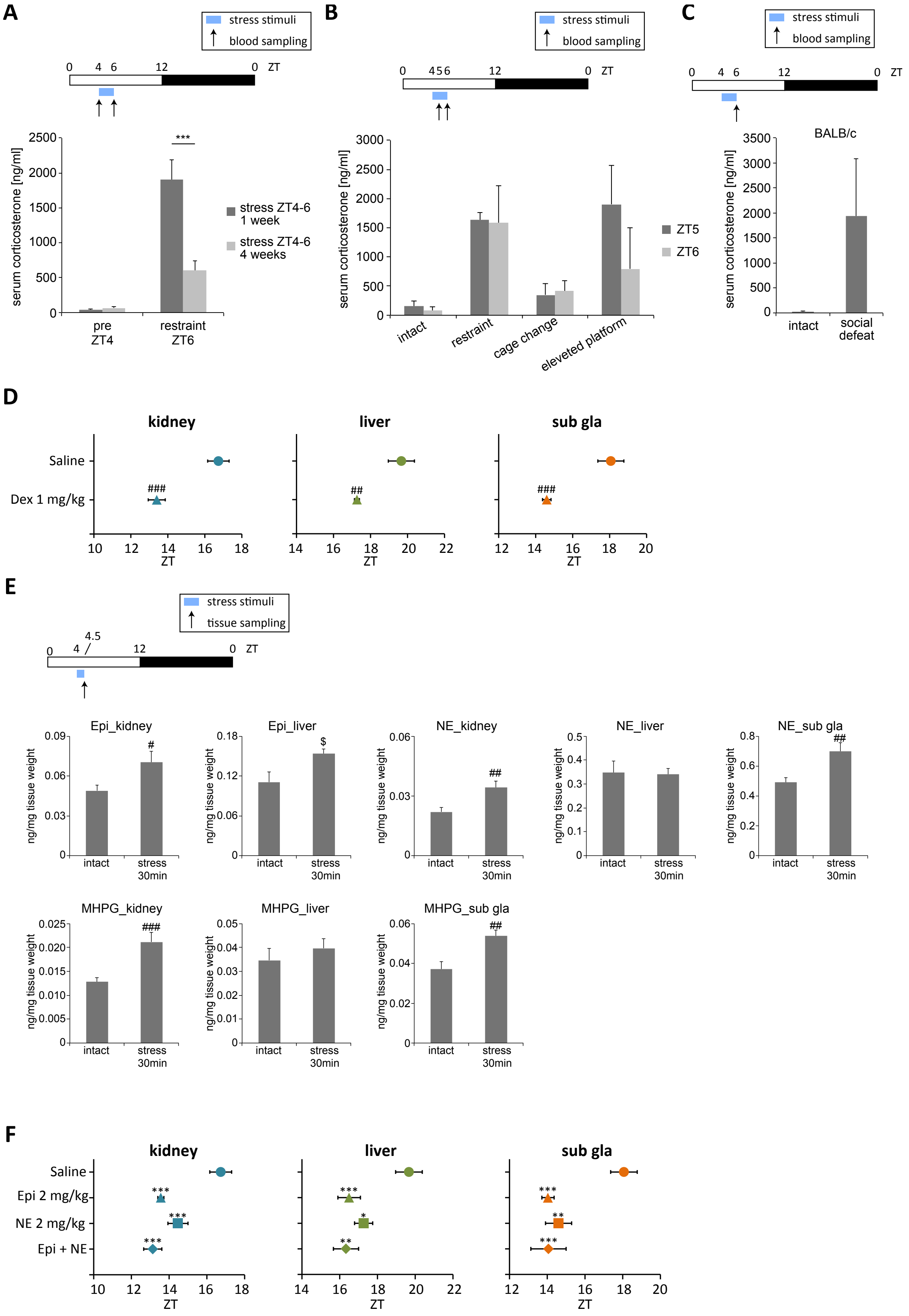

A
stress stimuli
blood sampling
0 4 6 12 0 ZT
serum corticosterone [ng/ml]
2500
2000
1500
1000
500
0
***
stress ZT4-6 1 week
stress ZT4-6 4 weeks
pre ZT4
restraint ZT6
B
stress stimuli
blood sampling
0 456 12 0 ZT
serum corticosterone [ng/ml]
3000
2500
2000
1500
1000
500
0
ZT5
ZT6
intact
restraint
cage change
eleveted platform
C
stress stimuli
blood sampling
0 4 6 12 0 ZT
BALB/c
serum corticosterone [ng/ml]
3500
3000
2500
2000
1500
1000
500
0
intact
social defeat
D
kidney
liver
sub gla
Saline
Dex 1 mg/kg
###
##
###
10 12 14 16 18
14 16 18 20 22
12 14 16 18 20
ZT
E
stress stimuli
tissue sampling
4.5
0 4 12 0 ZT
Epi_kidney
Epi_liver
NE_kidney
NE_liver
NE_sub gla
ng/mg tissue weight
0.09 0.06 0.03 0
0.18 0.12 0.06 0
0.06 0.03 0
0.5 0.4 0.3 0.2 0.1 0
0.8 0.6 0.4 0.2 0
#
$
##
##
intact
stress 30min
MHPG_kidney
MHPG_liver
MHPG_sub gla
0.025 0.020 0.015 0.010 0.005 0
0.05 0.04 0.03 0.02 0.01 0
0.06 0.04 0.02 0
###
##
F
kidney
liver
sub gla
Saline
Epi 2 mg/kg
NE 2 mg/kg
Epi + NE
***
*
**
10 12 14 16 18
14 16 18 20 22
12 14 16 18 20
ZT

Supplementary Figure S6. The roles of the HPA and SAM axes in stress-induced entrainment.

(A) Serum corticosterone levels in mice subjected to a single period or repeated periods of 3 days restraint stress (related to Figure 5A). Blood samples were collected at ZT4 and ZT6 on day 3 of stress exposure. $P < 0.01$, $F(1, 12) = 22.30$, for the interaction effect (two-way ANOVA). ***$P < 0.001$ (Sidak post hoc test). Values are expressed as mean ± SEM. N = 4 per group. (B, C) Levels of serum corticosterone in mice subjected to a single treatment of each stress exposure (related to Figure 6 and S7A). Serum was collected at ZT5 or ZT6 (B), and at ZT6 (C). Values are expressed as mean ± SEM. N = 4 per group, except in the elevated platform group where n = 3. (D) Peak phases of PER2::LUC oscillation in each tissue after injection of dexamethasone (Dex) (an analogue of corticosterone; 1 mg/kg, i.p.; Sigma-Aldrich, MO, USA) at ZT4 for 3 consecutive days. ##$P < 0.01$, ###$P < 0.001$ vs. saline (Student's t-test). (E) Catecholamine contents in peripheral tissues after 30 min of restraint stress by HPLC analysis. Epinephrine (Epi), norepinephrine (NE), and their metabolite (3-methoxy-4-hydroxyphenylglycol, MHPG) were measured. Values are expressed as mean + SEM. N = 9 for intact and n = 6 for restraint.

(F) Peak phases of PER2::LUC oscillation in each tissue after injection of epinephrine (Epi), norepinephrine (NE), or both (2 mg/kg, i.p.; Sigma-Aldrich, MO, USA) at ZT4 for 3 consecutive days. *$P < 0.05$, **$P < 0.01$, ***$P < 0.001$ vs. saline (one-way ANOVA with Dunnett test). Values are expressed as mean ± SEM.

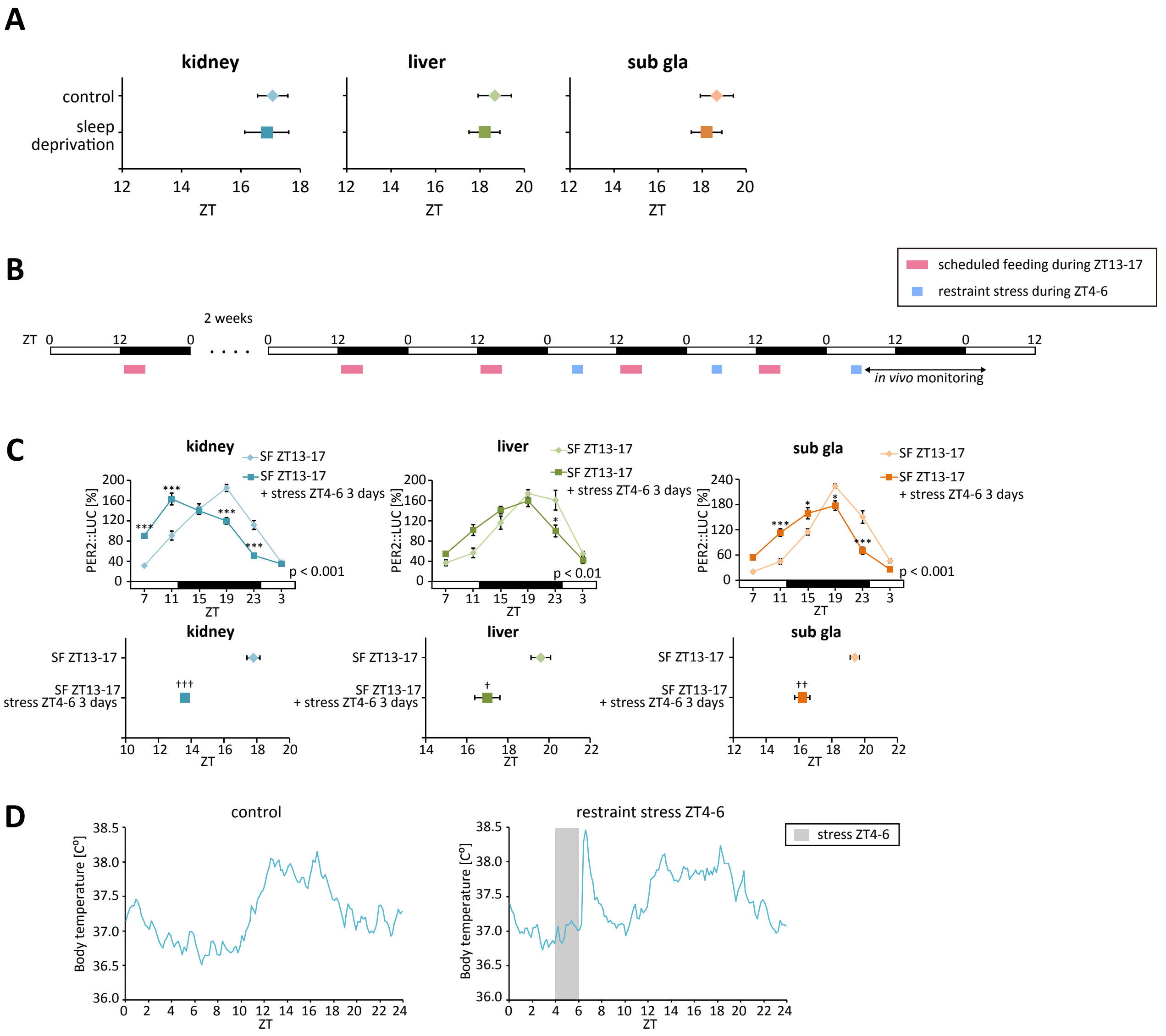

Supplementary Figure S7. Effects of sleep deprivation or activity increase, feeding behaviour, and body temperature on restraint stress-induced phase entrainment in peripheral tissues.

(A) Peak phases of PER2::LUC oscillation in each tissue after cage change stimulation (4 times at ZT4–6) for 3 days. Cage change stimulation increased activity and inhibited sleep, but the peripheral clocks were not responsive to these changes. (B) Experimental schedule for feeding and stress stimuli. After a 2-week period of adaptation to a scheduled feeding

paradigm (ZT13–17), mice were subjected to restraint stress at ZT4–6 for 3 days and PER2::LUC oscillations were monitored. Scheduled feeding was carried out using an automated feeding apparatus. (C) Waveforms and peak phases of PER2::LUC oscillations in each tissue. Increased activity was observed with lower intake of food after restraint stress (see Fig 5A), and the feeding behaviour in the light phase might cause phase-advance of the peripheral clocks. However, restraint-induced phase shift was observed even when feeding time was restricted to during the dark phase, suggesting that feeding behaviour is not involved in restraint stress-induced phase shift of the peripheral clocks. (D) Body temperature measured by intraperitoneal insertion of a temperature sensor (#3650, Hioki E.E. Co., Nagano, Japan). Values are expressed as mean ± SEM (n = 8 per group). P values shown on the lower right side of each graph indicate results of a two-way ANOVA between the control and stress groups. *P < 0.05, ***P < 0.001 vs. control (two-way ANOVA with Tukey post-hoc test); †P< 0.05, ††P < 0.01, †††P < 0.001 vs. control (Student's t-test).

Supplementary Table S1. Number of mice examined and tissues that met (passed) rhythmicity criteria in each experimental group.

group name		sex	strain	number of mice	number of tissue samples (pass/total)			Figure No.
					kidney	liver	sub gla	
control		female	PER2::LUC	6	6/6	6/6	6/6	1B, 1C, 2B, 4B, 4C, 5B,5D, S7A, 6A, S2B, S2C
restraint stress	1 day	female	PER2::LUC	3	3/3	3/3	3/3	2B
restraint stress	2 days	female	PER2::LUC	6	6/6	4/6	6/6	2B
restraint stress	3 days	female	PER2::LUC	6	6/6	4/6	6/6	1B, 1C, 2B, 4A-C, 5B, S2C
restraint stress	4 days	female	PER2::LUC	5	5/5	5/5	5/5	2B
restraint stress	5 days	female	PER2::LUC	3	3/3	3/3	3/3	2B
restraint stress	6 days	female	PER2::LUC	3	3/3	3/3	3/3	2B
restraint stress	7 days	female	PER2::LUC	6	6/6	6/6	6/6	2B
restraint stress	14 days	female	PER2::LUC	6	4/6	6/6	6/6	2B
restraint stress	3 days + 24 hr	female	PER2::LUC	4	4/4	4/4	4/4	S2B, C
restraint stress	ZT0-2	female	PER2::LUC	8	1/8	5/8	5/8	4A-D
restraint stress	ZT12-14	female	PER2::LUC	4	4/4	4/4	4/4	4A-C
restraint stress	ZT16-18	female	PER2::LUC	4	4/4	4/4	4/4	4A-C
restraint stress	ZT20-22	female	PER2::LUC	5	5/5	4/5	5/5	4A-C
control		male	PER2::LUC	4	3/4	4/4	4/4	S1B-D
restraint stress	3 days	male	PER2::LUC	3	3/3	3/3	3/3	S1B-D
sleep deprivation		female	PER2::LUC	3	3/3	3/3	3/3	S7A
elevated platform		female	PER2::LUC	3	3/3	3/3	3/3	6A
control		male	*Bmal1-Eluc*	6	5/6	5/6	6/6	S5A, S5B, 6B
restraint stress	3 days	male	*Bmal1-Eluc*	3	3/3	3/3	3/3	S5A, S5B
social defeat		male	*Bmal1-Eluc*	6	5/6	6/6	4/6	6B
restraint stress	ZT4-6 3 days, 4weeks	female	PER2::LUC	9	8/9	9/9	9/9	5B
control		female	PER2::LUC	3	3/3	3/3	3/3	5D
restraint stress	ZT0-2 3 days, 5weeks	female	PER2::LUC	3	3/3	3/3	3/3	5D
SF ZT13-17		female	PER2::LUC	4	4/4	4/4	4/4	S7C
SF ZT13-17 + restraint stress		female	PER2::LUC	4	4/4	3/4	4/4	S7C
saline		female	PER2::LUC	6	6/6	6/6	6/6	S6D, S6F
Dex 1 mg/kg		female	PER2::LUC	3	3/3	3/3	3/3	S6D
Epi 2 mg/kg		female	PER2::LUC	5	5/5	4/5	5/5	S6F
NE 2 mg/kg		female	PER2::LUC	3	3/3	3/3	3/3	S6F
Epi + NE		female	PER2::LUC	3	3/3	3/3	3/3	S6F

SF, scheduled feeding; sub gla, submandibular gland; ZT, Zeitgeber time.

Supplementary Table S2. Statistical analyses of data analysed with one- or two-way ANOVA.

Figure No.	detail	ANOVA	main effect A			main effect B			Interaction effect	
			factor	F	P-value	factor	F	P-value	F	P-value
1C	kidney	two-way repeated measures	time	F (5, 50) = 44.20	p < 0.001	group	F (1, 10) = 1.00	p = 0.340	F = (5, 50) = 22.97	p < 0.001
1C	liver	two-way repeated measures	time	F (5, 40) = 25.74	p < 0.001	group	F (1, 8) = 0.64	p = 0.446	F = (5, 40) = 7.52	p < 0.001
1C	sub gla	two-way repeated measures	time	F (5, 50) = 59.42	p < 0.001	group	F (1, 10) = 1.00	p = 0.340	F (5, 50) = 31.73	p < 0.001
1D	*Per2*	two-way	time	F (5, 24) = 22.23	p < 0.001	group	F (1, 24) = 1.56	p = 0.223	F (5, 24) = 14.59	p < 0.001
1E	*Per1*	two-way	time	F (5, 24) = 4.95	p < 0.01	group	F (1, 24) = 0.38	p = 0.538	F (5, 24) = 5.18	p < 0.01
1E	*Per2*	two-way	time	F (5, 24) = 5.91	p < 0.01	group	F (1, 24) = 0.22	p = 0.639	F (5, 24) = 5.00	p < 0.01
1F	*Per1*	two-way	time	F (5, 24) = 4.49	p < 0.01	group	F (1, 24) = 5.00	p < 0.05	F (5, 24) = 4.47	p < 0.01
1F	*Per2*	two-way	time	F (5, 24) = 7.03	p < 0.001	group	F (1, 24) = 0.09	p = 0.762	F (5, 24) = 11.08	p < 0.001
2B	kidney	one-way	group	F (8, 33) = 19.94	p < 0.001					
2B	liver	one-way	group	F (8, 31) = 10.02	p < 0.001					
2B	sub gla	one-way	group	F (8, 35) = 20.61	p < 0.001					
5B	kidney (upper)	two-way repeated measures	time	F (5, 60) = 87.63	p < 0.001	group	F (1, 12) = 0.05	p = 0.818	F (5, 60) = 18.10	p < 0.001
5B	liver (upper)	two-way repeated measures	time	F (5, 55) = 52.01	p < 0.001	group	F (1, 11) = 1.31	p = 0.275	F (5, 55) = 9.27	p < 0.001
5B	sub gla (upper)	two-way repeated measures	time	F (5, 65) = 105.9	p < 0.001	group	F (1, 13) = 0.78	p = 0.392	F (5, 65) = 12.27	p < 0.001
5B	kidney (lower)	one-way	group	F (2, 17) = 21. 50	p < 0.001					
5B	liver (lower)	one-way	group	F (2, 16) = 8.12	p < 0.01					
5B	sub gla (lower)	one-way	group	F (2, 18) = 25.30	p < 0.001					
6B	kidney	two-way repeated measures	time	F (5, 40) = 38.68	p < 0.001	group	F (1, 8) = 0.65	p = 0.443	F (5, 40) = 9.21	p < 0.001
6B	liver	two-way repeated measures	time	F (5, 45) = 45.10	p < 0.001	group	F (1, 9) = 0.67	p = 0.431	F (5, 45) = 3.74	p < 0.01
6B	sub gla	two-way repeated measures	time	F (5, 40) = 26.81	p < 0.001	group	F (1, 8) = 0.11	p = 0.741	F (5, 40) = 8.44	p < 0.001
S1C	kidney	two-way repeated measures	time	F (5, 20) = 36.84	p < 0.001	group	F (1, 4) = 0.04	p = 0.844	F (5, 20) = 11.74	p < 0.001
S1C	liver	two-way repeated measures	time	F (5, 25) = 32.74	p < 0.001	group	F (1, 5) = 0.43	p = 0.538	F (5, 25) = 11.94	p < 0.001
S1C	sub gla	two-way repeated measures	time	F (5, 25) = 7.88	p < 0.001	group	F (1, 5) = 7.88	p < 0.05	F (5, 25) = 37.83	p < 0.001
S2B	kidney	two-way repeated measures	time	F (5, 40) = 41.74	p < 0.001	group	F (1, 8) = 0.64	p = 0.743	F (5, 40) = 7.05	p < 0.001
S2B	sub gla	two-way repeated measures	time	F (5, 40) = 62.31	p < 0.001	group	F (1, 8) = 0.64	p = 0.446	F (5, 40) = 8.62	p < 0.001
S2C	kidney	one-way	group	F (2, 13) = 29.96	p < 0.001					
S2C	liver	one-way	group	F (2, 11) = 8.71	p < 0.01					
S2C	sub gla	one-way	group	F (2, 13) = 42.67	p < 0.001					
S4A	kidney	two-way repeated measures	time	F (5, 30) = 28.49	p < 0.001	group	F (1, 6) = 1.31	p < 0.001	F (5, 30) = 7.03	p < 0.001
S4A	sub gla	two-way repeated measures	time	F (5, 35) = 19.85	p < 0.001	group	F (1, 7) = 1.81	p = 0.22	F (5, 35) = 6.58	p < 0.001
S6C	kidney	two-way repeated measures	time	F (5, 30) = 75.61	p < 0.001	group	F (1, 6) = 0.13	p = 0.726	F (5, 30) = 28.26	p < 0.001
S5C	liver	two-way repeated measures	time	F (5, 25) = 35.11	p < 0.001	group	F (1, 5) = 0.01	p = 0.905	F (5, 25) = 5.07	p < 0.01
S5C	sub gla	two-way repeated measures	time	F (5, 30) = 90.30	p < 0.001	group	F (1, 6) = 0.33	p = 0.582	F (5, 30) = 18.76	p < 0.001
S6F	kidney	one-way	group	F (3, 13) = 28.87	p < 0.001					
S6F	liver	one-way	group	F (3, 12) = 12.50	p < 0.001					
S6F	sub gla	one-way	group	F (3, 13) = 15.26	p < 0.001					

Supplementary Table S3. Cosinor analysis of RT-PCR data.

Figure No.	Tissue	Gene name	Group	Goodness of fit	Achrophase (hr)	Phase shift vs. control (hr)
1D	kidney	*Per1*	control	0.05	10.6	
1D	kidney	*Per1*	stress	0.021	7	−3.6
1D	kidney	*Per2*	control	0.018	13	
1D	kidney	*Per2*	stress	0.006	7.8	−5.2
1D	kidney	*Bmal1*	control	0.024	19	
1D	kidney	*Bmal1*	stress	0.042	15.8	−3.2
1D	kidney	*Dbp*	control	0.04	7.8	
1D	kidney	*Dbp*	stress	0.064	3	−4.8
1D	kidney	*Rev-erbα*	control	0.07	3	
1D	kidney	*Rev-erbα*	stress	0.015	0.6	−2.4
1E	hippocampus	*Per1*	control	0.059	13	
1E	hippocampus	*Per1*	stress	0.054	7	−6
1E	hippocampus	*Per2*	control	0.053	13	
1E	hippocampus	*Per2*	stress	0.041	7.8	−5.2
1F	cortex	*Per1*	control	0.354	(12.2)	
1F	cortex	*Per1*	stress	0.051	7	(−5.2)
1F	cortex	*Per2*	control	0.084	14.6	
1F	cortex	*Per2*	stress	0.045	7.4	−7.2
4E	kidney	*Per1*	stress	5.45	(3)	
4E	kidney	*Per2*	stress	0.271	(3)	
4E	kidney	*Bmal1*	stress	0.029	13	−6
4E	kidney	*Rev-erbα*	stress	0.063	20.2	−6.8
4E	kidney	*Dbp*	stress	0.011	23	−8.8
S1A	liver	*Per1*	control	0.026	11.4	
S1A	liver	*Per1*	stress	0.035	9	−2.4
S1A	liver	*Per2*	control	0.016	14.2	
S1A	liver	*Per2*	stress	0.059	10.2	−4
S1A	liver	*Bmal1*	control	0.024	20.6	
S1A	liver	*Bmal1*	stress	0.046	18.6	−2
S1A	liver	*Dbp*	control	0.066	8.6	
S1A	liver	*Dbp*	stress	0.216	(3)	(−5.6)
S1A	liver	*Rev-erbα*	control	0.042	7	
S1A	liver	*Rev-erbα*	stress	0.066	1	−6
S1A	sub gla	*Per1*	control	0.152	(10.2)	
S1A	sub gla	*Per1*	stress	0.041	7.4	(−2.8)
S1A	sub gla	*Per2*	control	0.025	13	
S1A	sub gla	*Per2*	stress	0.006	9.8	−3.2
S1A	sub gla	*Bmal1*	control	0.006	22.2	
S1A	sub gla	*Bmal1*	stress	0.072	18.2	−4
S1A	sub gla	*Dbp*	control	0.061	8.2	
S1A	sub gla	*Dbp*	stress	0.102	(3)	(−5.2)
S1A	sub gla	*Rev-erbα*	control	0.051	7	
S1A	sub gla	*Rev-erbα*	stress	0.017	2.2	−4.8
S1A	adrenal	*Per1*	control	0.102	(8.6)	
S1A	adrenal	*Per1*	stress	0.205	(7)	(−1.6)
S1A	adrenal	*Per2*	control	0.016	11	
S1A	adrenal	*Per2*	stress	0.007	7.8	−3.2
S1A	adrenal	*Bmal1*	control	0.026	18.2	
S1A	adrenal	*Bmal1*	stress	0.035	13	−5.2
S1A	adrenal	*Dbp*	control	0.085	7	
S1A	adrenal	*Dbp*	stress	0.059	1	−6
S1A	adrenal	*Rev-erbα*	control	0.073	3	
S1A	adrenal	*Rev-erbα*	stress	0.053	23.8	−3.2

Goodness of fit values (<0.1) represent rhythmic oscillation. The achrophase and phase difference values in parenthesis are arrhythmic oscillations.

Supplementary Table S4. Primer sequences for RT-PCR analysis.

Gapdh	5'-tggtgaaggtcggtgtgaac-3'
	5'-aatgaaggggtcgttgatgg-3'
Per1	5'-caagtggcaatgagtccaacg-3'
	5'-cgaagtttgagctcccgaagtg-3'
Per2	5'-tgtgtgcttacacgggtgtccta-3'
	5'-acgtttggtttgcgcatgaa-3'
Bmal1	5'-ccacctcagagccattgataca-3'
	5'-gagcaggtttagttccactttgtct-3'
Cry1	5'-atccgctgcgtctatatcctc-3'
	5'-cccgaatcacaaacagacg-3'
Dbp	5'-ccgtggaggtgctaatgacct-3'
	5'-cctctgagaagcggtgcct-3'
Rev-erbα	5'-cttccgtgacctttctcagc-3'
	5'-cagctcctcctcggtaagtg-3'
